# The effect of exercise on cerebral blood flow and executive function among young adults: a double-blinded randomized controlled trial

**DOI:** 10.1038/s41598-023-33063-9

**Published:** 2023-05-22

**Authors:** Jianxiu Liu, Leizi Min, Ruidong Liu, Xiaoyu Zhang, Meiting Wu, Qian Di, Xindong Ma

**Affiliations:** 1 Vanke School of Public Health, Tsinghua University, Beijing, 100084 China; 2 Division of Sports Science and Physical Education, Tsinghua University, Beijing, 100084 China; 3 Sports Coaching College, Beijing Sport University, Beijing, 100084 China; 4 AME2P Laboratory, Clermont Auvergne University, 63178 Clermont-Ferrand, France; 5 Department of Physical Education, Zhejiang College of Sports, Hangzhou, 310013 Zhejiang China; 6 Institute for Healthy China, Tsinghua University, Beijing, 100084 China; 7 IDG/McGovern Institute for Brain Research, Tsinghua University, Beijing, 100084 China

**Keywords:** Health care, Public health

## Abstract

Studies have demonstrated that exercise benefits executive function. However, it remains unclear which type of exercise is optimal for preserving executive function among young adults and the cerebral blood flow (CBF) mechanisms that underlie exercise-induced cognitive benefits. Therefore, this study aims to compare the intervention effects of high-intensity interval training (HIIT) and moderate-intensity continuous training (MICT) on executive function and the CBF mechanism. This was a double-blinded, randomized, controlled trial study conducted between October 2020 and January 2021 (ClinicalTrials.gov identifier: NCT04830059). Ninety-three healthy young adults (25.23 ± 2.18 years old; 49.82% male) were randomized into the HIIT (N = 33), MICT (N = 32), and control (N = 28) groups. Participants in exercise groups were guided to perform 40 min of HIIT and MICT three times a week for 12 weeks, while the control group received health education for the same period. The primary outcomes, changes in executive function assessed by the trail-making test (TMT), and CBF measured by transcranial Doppler flow analyzer (EMS-9WA), were evaluated before and after the interventions. The time taken to complete the TMT task improved significantly in the MICT group compared to the control group [β = −10.175, 95%, confidence interval (CI) =  −20.320, −0.031]. Additionally, the MICT group showed significant improvements in the pulsatility index (PI) (β = 0.120, 95% CI = 0.018, 0.222), resistance index (RI) (β = 0.043, 95% CI = 0.005, 0.082), and peak-systolic/end-diastolic velocity (S/D) (β = 0.277, 95% CI = 0.048, 0.507) of CBF compared to the control group. The time taken to complete the TMT was associated with the velocity of peak-systolic (F = 5.414, *P* = 0.022), PI (F = 4.973, *P* = 0.012), and RI (F = 5.845, *P* = 0.006). Furthermore, the accuracy of TMT was associated with PI (F = 4.797, *P* = 0.036), RI (F = 5.394, *P* = 0.024), and S/D (F = 4.312, *P* = 0.05) of CBF. A 12-week MICT intervention improved CBF and executive function more effectively than HIIT among young adults. Furthermore, the findings suggest that CBF was one of the potential mechanisms underlying the cognitive benefits of exercise in young people. These results provide practical evidence supporting the promotion of regular exercise to maintain executive function and improve brain health.

## Introduction

Studies have reported the positive effect of exercise on the musculoskeletal system^1^, cardiovascular system^2^, respiratory system^3^, and other physical functions^4^. However, the benefits of exercise extend beyond physical health, and physically active individuals exhibit better neurocognitive function than inactive individuals^5^. Executive function is a central component of cognition, known to influence various cognitive processes relevant to many domains of daily living, including academic achievement^4^, vocational performance^6^, and quality of life^7^. The effects of exercise training on cognitive function are reported to be the largest for executive function compared to other cognitive domains^8^. Regular exercise improves executive function among adolescents, older adults, and even patients with cognitive impairments^9,10^. Prior studies confirmed that acute exercise could enhance working memory, processing speed, attention, and inhibition^11,12^. Limited research has explored the potential impact of chronic exercise on memory and cognitive control among young adults^13–15^. Nonetheless, further investigation is required to confirm the benefits of chronic exercise on executive function, particularly among young adults whose cognitive capacity is at its peak.

Aerobic exercise, including moderate-intensity continuous training (MICT), can enhance cognitive performance throughout the life span^16^. The available evidence emphasizes the significance of promoting physical activity across the lifespan to enhance cognitive abilities, counteract neural degeneration, and prevent neurodegenerative disease^17–19^. Over the past few years, a mounting body of evidence has demonstrated the efficiency of high-intensity interval training (HIIT), which involves alternating high-intensity exercise bouts with periods of rest (e.g., 30 s of high-intensity activity followed by 30 s of rest, repeated for 5 min), for enhancing physical and psychological health-related outcomes^20,21^. Compared to aerobic exercise, HIIT can achieve similar physiological adaptations within a shorter time frame^22^. Several studies have indicated that HIIT leads to greater improvements in cardiorespiratory fitness and vascular function compared to MICT^23,24^. Moreover, HIIT has been shown to enhance memory performance to a greater extent than MICT among sedentary elderly individuals^25^. HIIT has been shown to increase the production of lactic acid in the body, which can serve as an energy substrate in the brain and support brain metabolism^26^. Furthermore, lactic acid has been found to play a pivotal role in long-term potentiation and improvements in cognitive function^27^. However, few studies compared the effects of different modalities of chronic exercise on executive function, especially the cognitive benefit of exercise among young adults^28^. Such studies would be valuable in guiding physical activity recommendations for this population.

Despite accounting for only 2% of the human body mass, the brain consumes approximately one-fifth of the cardiac output, making it the most metabolically active organ in the human body^29^. Maintaining a relatively constant cerebral blood flow (CBF) is fundamental to preserving normal brain function and metabolic activities^30^. Previous research has suggested that no effect of acute exercise on CBF or cerebrovascular reactivity in the intracranial or extracranial arteries among young adults, indicating that CBF is rapidly and uniformly regulated after acute exercise^31^. However, evidence has shown that exercise training can alter CBF in older adults, with or without cognitive impairment^32^, and that the decrease in CBF could be mitigated by two-minute light-intensity walking breaks every 30 min^33^. Research indicated that one week of aerobic and resistance exercise training could selectively increase the blood flow in hippocampal regions^34^. In terms of the effect of exercise on CBF and cognition, evidence has demonstrated that CBF regulation partially mediates the link between physical activity and cognition in older adults, and that improvements in CBF regulation are associated with better cognitive functioning^35^. Specifically, increased middle cerebral artery blood velocity has been identified as a potential mechanism through which exercise can prevent cerebrovascular and neurological diseases^36^. Animal studies also revealed that exercise could increase the number of neurons in the hippocampus of rats with bilateral common carotid artery embolism and delay cognitive decline by promoting neurogenesis and enhancing the expression of brain-derived neurotrophic factor^37^. Exercise increases the production of neurotrophic and vascular growth factors, which promote new growth and plasticity and help maintain the structural integrity of the brain and cerebrovascular system^23^. This mechanism may help explain the effect of exercise on CBF. Thus, it is plausible to hypothesize that exercise training could enhance the executive function of young individuals by improving cerebral blood flow.

Therefore, despite the common assumption that cognitive functioning is at its peak in healthy young adults, recent research has indicated a positive association between habitual physical activity and cognitive performance^38^. Nevertheless, few studies have investigated the effects of exercise interventions on cognitive function and CBF in young individuals, and evidence is lacking regarding the potential CBF mechanisms underlying exercise-related improvements in executive function. Therefore, the present study aims to (1) compare the effects of 12 weeks of MICT and HIIT on cognitive function in young adults, and (2) investigate the potential role of cerebral blood flow mechanisms in the exercise-induced improvements in executive function.

## Results

### Descriptive analysis of the demographic information

The descriptive analysis of the study population is indicated in Table 1. Sixty-seven subjects were included in the data analysis (HIIT = 26, MICT = 21, control group = 20), and all participants were analyzed according to the initial grouping. There were no significant differences among groups in the demographic information, including age, height, Body Mass Index (BMI), years of education, and birthplace. No significant difference was found in baseline maximum oxygen uptake (VO_2 max_) between groups. Overall, 49.82% of the study population was male. Participants across all three groups were 25.23 ± 2.18 years old on average, with a mean BMI of 21.91 ± 2.92 kg/m^2^. The mean VO_2 max_ of all the participants was 43.71 ± 6.98 mL/(kg·min). No significant differences were found in subjective fatigue and enjoyment between the MICT and HIIT groups, as measured by the rating of perceived exertion scale (RPE) and the physical activity enjoyment scale (PACES) (Supplementary Table S1). Furthermore, both the HIIT and MICT groups showed a decrease in blood pressure (systolic and diastolic) and resting heart rate after the intervention compared to the control group (Supplementary Table S2). However, no association was found between blood pressure and CBF.

**Table 1:** Demographic information of participants.

		MICT (N = 21)	HIIT (N = 26)	CON (N = 20)	*F/X*^*2*^	*P*
Age (years)		25.44 ± 1.58	24.83 ± 2.35	25.42 ± 2.61	0.74	0.48
Height (cm)		169.53 ± 7.69	169.67 ± 6.47	168.89 ± 7.35	0.98	0.39
Weight (kg)		65.53 ± 14.25	62.99 ± 10.25	60.44 ± 10.79	1.29	0.28
BMI (kg/m^2^)		22.42 ± 3.31	21.75 ± 2.49	21.55 ± 2.96	0.73	0.49
Years of education (from freshman)		6.78 ± 1.36	6.10 ± 1.47	7.23 ± 2.41	2.94	0.06
Birthplace (urban%)		19 (90.48)	21 (80.77)	13 (65.00)	4.09	0.13
Gender (male%)		12 (57.14)	11 (42.31)	10 (50.00)	1.03	0.60
VO_2_max [mL/(kg·min)]	Time1	42.31 ± 6.00	43.90 ± 7.63	44.92 ± 7.01	1.00	0.37
	Time2	44.24 ± 6.11	45.65 ± 6.74	44.43 ± 6.80	0.42	0.66
Systolic pressure	Time1	118.74 ± 12.67	119.36 ± 10.94	114.50 ± 12.13	1.24	0.30
	Time2	118.67 ± 12.49	116.37 ± 11.69	122.28 ± 14.89	1.43	0.25
Diastolic pressure	Time1	71.63 ± 9.20	70.95 ± 8.60	69.03 ± 5.84	0.72	0.49
	Time2	69.67 ± 7.48	68.57 ± 8.79	70.72 ± 7.36	0.50	0.61
Rest heart rate	Time1	72.52 ± 10.94	71.59 ± 13.34	73.07 ± 10.24	0.10	0.90
	Time2	69.70 ± 9.50	68.10 ± 10.30	76.84 ± 11.07	5.41	0.006
TMT-ACC	Time1	0.93 ± 0.08	0.95 ± 0.08	0.96 ± 0.01	0.76	0.47
	Time2	0.98 ± 0.04	0.98 ± 0.04	0.96 ± 0.05	1.10	0.34
TMT-TIME (s)	Time1	96.14 ± 15.46	93.54 ± 19.47	98 ± 22.99	0.30	0.74
	Time2	78.80 ± 14.06	82.77 ± 16.34	90.84 ± 17.35	2.93	0.06†
Vs (cm/s)	Time1	92.29 ± 16.22	94.19 ± 11.59	96.35 ± 14.97	0.42	0.66
	Time2	95.38 ± 18.23	95.92 ± 11.71	95.15 ± 13.89	0.02	0.98
Vd (cm/s)	Time1	40.90 ± 6.77	43.04 ± 8.19	42.85 ± 7.82	0.52	0.60
	Time2	39.29 ± 8.80	42.08 ± 6.67	43.40 ± 7.48	1.58	0.21
Vm (cm/s)	Time1	58.05 ± 9.51	60.04 ± 8.92	60.60 ± 9.81	0.43	0.65
	Time2	57.95 ± 11.45	60.04 ± 7.39	60.45 ± 9.10	0.44	0.65
PI	Time1	0.88 ± 0.11	0.86 ± 0.14	0.88 ± 0.12	0.19	0.83
	Time2	0.98 ± 0.18*	0.90 ± 0.16	0.86 ± 0.12	3.25	0.04*
RI	Time1	0.55 ± 0.05	0.54 ± 0.06	0.55 ± 0.05	0.27	0.77
	Time2	0.59 ± 0.06*	0.56 ± 0.06	0.54 ± 0.05	3.16	0.04*
S/D	Time1	2.26 ± 0.22	2.23 ± 0.27	2.27 ± 0.23	0.14	0.87
	Time2	2.49 ± 0.44*	2.31 ± 0.34	2.21 ± 0.24	3.17	0.04*

*MICT* moderate-intensity continuous training, *HIIT* high-intensity interval training, *CON* control, *TMT* trail-making test, *Vs* velocity of peak-systolic, *Vd* velocity of end-diastolic, *Vm* velocity of mean cerebral blood flow, *PI* pulsatility index, *RI* resistance index, *S/D* peak-systolic of cerebral blood flow/end-diastolic of cerebral blood flow.

*Significant difference with the control group (*p* < 0.05).

### Effects of exercise intervention on executive function

**Figure 1:**
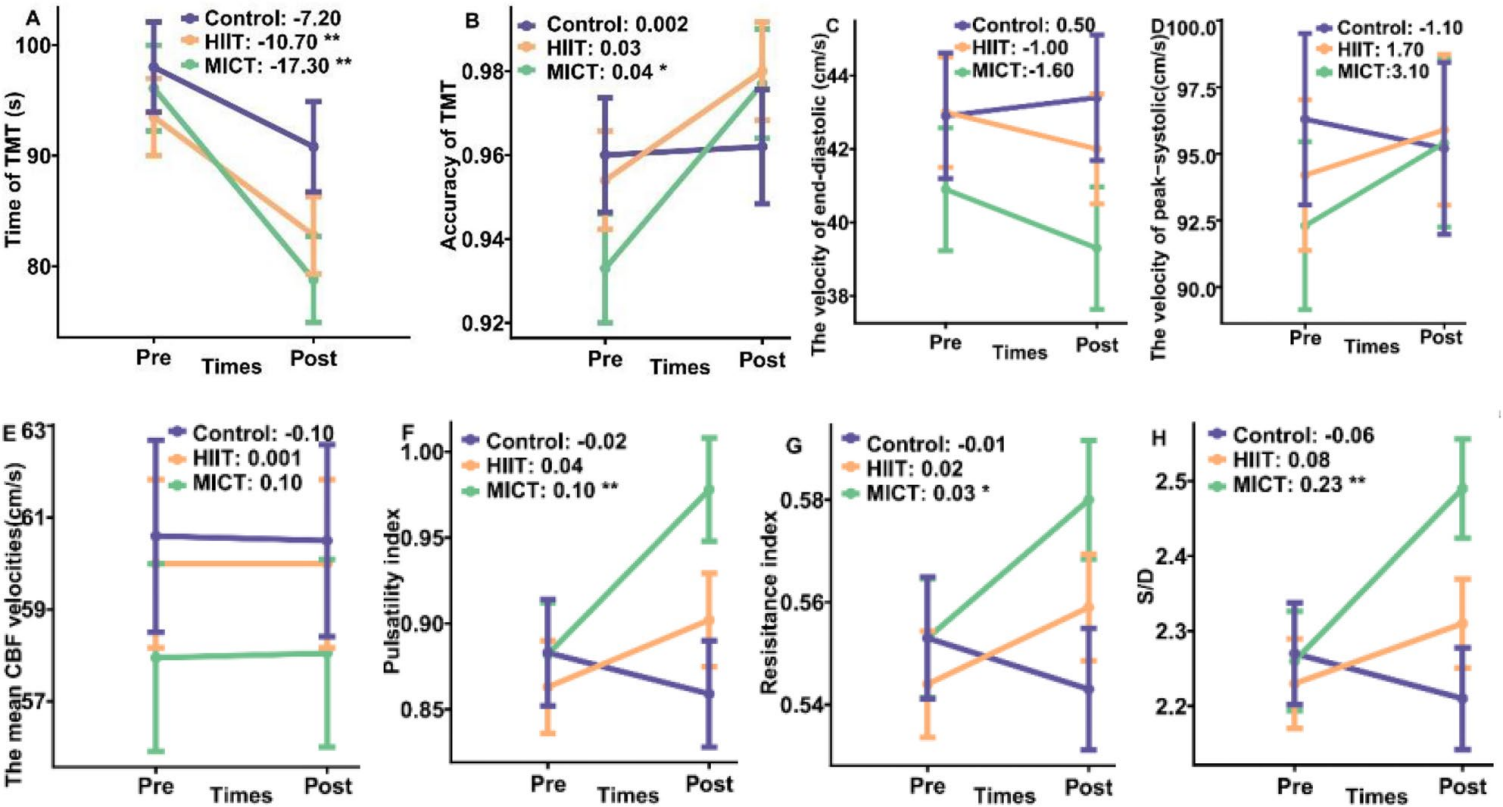
The effect of pre-post effect after 12-week intervention on cognitive performance and CBF. Executive function and CBF indicators measured in pre-post of 12-week exercise interventions. *MICT* moderate-intensity continuous training, *HIIT* high-intensity interval training. The estimated values of pairwise analysis between pre and post-measurement in different groups were shown in the subgraph (**A–H**). Asterisk: a statistically significant difference between groups during the pre-post intervention period. *p < 0.05; **p < 0.01.

The cognitive differences between groups after three months of intervention indicated that the time of the TMT task among the MICT group improved significantly compared with the control group (β = −10.175, 95% CI = −20.320, −0.031). No significant difference was found in reaction time (RT) when comparing the HIIT vs. control groups and HIIT vs. MICT groups (Table 2). The reaction time of the TMT task improved significantly among MICT (estimated value = −17.34, *P* < 0.01) and HIIT (estimated value = −10.77, *P* < 0.01) groups after the 12-week intervention (Fig. 1 and Supplementary Table S4). Moreover, the accuracy of the TMT task improved in the MICT group (estimated value = 0.04,* P* < 0.05). The intra-group and inter-group comparisons indicated that three months of MICT and HIIT intervention improved the executive function of young adults, and MICT is more beneficial.

**Table 2:** Differences between groups of changes in executive function and cerebral blood flow.

Variable	Differences, post vs. pre, HIIT vs. control^a^	Difference, post vs. pre, MICT vs. control	Difference, post vs. pre, MICT vs. HIIT
	β (95% CI)	β (95% CI)	β (95% CI)
RT (ms)	−3.611 (−13.281, 6.059)	−**10.175 (**−**20.320, **−0.031)*	6.564 (−2.836, 15.964)
ACC	0.024 (−0.024, 0.072)	0.042 (−0.009, 0.092)	−0.018 (−0.064, 0.029)
Vs (cm/s)	2.931 (−5.233, 11.094)	4.295 (−4.280, 12.871)	−1.364 (−9.417, 6.688)
Vd (cm/s)	−1.512 (−6.656, 3.633)	−2.169 (−7.574, 3.236)	0.658 (−4.418, 5.733)
Vm (cm/s)	0.150 (−5.605, 5.905)	0.055 (−5.991, 6.101)	0.095 (−5.582, 5.773)
PI	0.063 (−0.034, 0.16)	0.120 (0.018, 0.222)*	−0.057 (−0.153, 0.038)
RI	0.025 (−0.012, 0.062)	0.043 (0.005, 0.082)*	−0.018 (−0.055, 0.018)
S/D	0.133 (−0.085, 0.352)	0.277 (0.048, 0.507)*	−0.144 (−0.360, 0.072)

Statistically significant results at 0.05 level are presented in bold.

*β* interactive effect estimates of mixed effect model, indicating the between-group difference during the 12-week intervention period, *CI* confidence interval, *MICT* moderate-intensity continuous training, *HIIT* high-intensity interval training, *RT* reaction time of TMT, *ACC* accuracy of TMT, *Vs* velocity of peak-systolic, *Vd* velocity of end-diastolic, *Vm* velocity of mean cerebral blood flow, *PI* pulsatility index, *RI* resistance index, *S/D* peak-systolic of cerebral blood flow/end-diastolic of cerebral blood flow.

^a^Differences between groups in change from pre to post-test, calculated via mixed effect models adjusted for gender, age, education level, body mass index, and birthplace, with a random effect on individuals to account for intrapersonal variation. ^a^The calculation formula that (post_MICT_ – pre_MICT_) – (post_control_ – pre_control_).

### Effects of exercise intervention on cerebral blood flow

The coefficient of variation of the CBF was shown in Supplementary Table S3. The pulsatility index (PI) (estimated value = 0.10, *P* < 0.01), resistance index (RI) (estimated value = 0.03, *P* < 0.01), and peak-systolic of cerebral blood flow/end-diastolic of cerebral blood flow (S/D) (estimated value = 0.23, *P* < 0.01) increased significantly among the MICT group after three months of intervention (Fig. 1 and Supplementary Table S4). The cognitive difference between groups after 3 months of the intervention indicated that the PI (β = 0.120, 95% CI = 0.018, 0.222), RI (β = 0.043, 95% CI = 0.005, 0.082), and S/D (β = 0.277, 95% CI = 0.048, 0.507) of the CBF among MICT group improved significantly compared with the control group (Table 2). We did not observe a significant change in the CBF index among the HIIT group after 3 months of intervention compared with the control group.

### Associations between the cerebral blood flow and executive function

**Figure 2:**
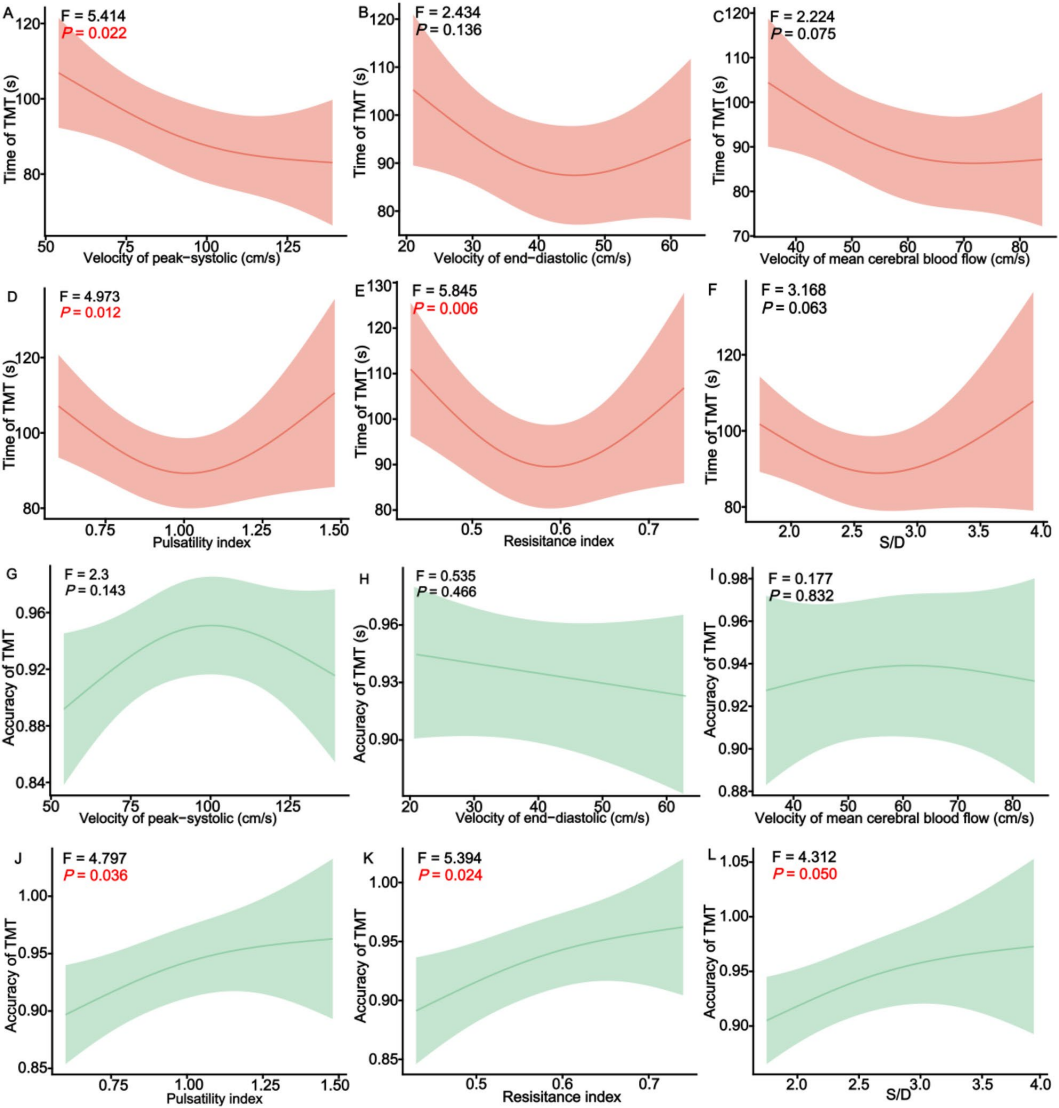
The dose–response associations between the executive function and CBF. *TMT* trail-making test, *S/D* peak-systolic of cerebral blood flow/end-diastolic of cerebral blood flow. Dose–response relationships were estimated by the generalized additive model with the penalized spline.

To assess dose–response and possible nonlinearity, we plotted bivariate associations of executive function and CBF using generalized additive models with the penalized spline. The results showed that the association of the accuracy of TMT with PI, RI, and S/D was approximately linear, while the time of TMT and CBF index were non-linear. (Fig. 2 and Supplementary Table S5). The time of TMT was associated with the velocity of peak-systolic (Vs) (F = 5.414, *P* = 0.022), PI (F = 4.973, *P* = 0.012), and RI (F = 5.845, *P* = 0.006). Moreover, the accuracy of TMT was associated with PI (F = 4.797, *P* = 0.036), RI (F = 5.394, *P* = 0.024), and S/D (F = 4.312, *P* = 0.05). The results indicated that the Vs, PI, and RI of CBF were associated with the time of TMT, and the PI, RI, and S/D of CBF were associated with the accuracy of TMT.

## Materials and methods

### Study design and population

We conducted a double-blind, randomized controlled trial (RCT) study from October 2020 to January 2021 using randomization, assignment concealment, and blinding methods (ClinicalTrials.gov identifier: NCT04830059. Data: 02/04/2021). Ninety-three participants met the inclusion criteria and were randomized into three groups: HIIT (N = 32), MICT (N = 33), and control (N = 28). Demographic information was collected, including age, gender, height, weight, family income, education level, and birthplace (rural or urban). Each participant had a unique identification number. They were randomly divided into different groups using a random number generator. The randomization process was carried out by a research staff member who did not participate in the assessment and analysis of the data. Group allocation was confidential to ensure the successful implementation of randomization. The participants’ identification number and grouping information were hidden in an opaque envelope, which was opened by themselves after the baseline assessment. Moreover, participants in the three groups came to the fitness centre on different days of the week, so they did not know the existence of other groups. Participants and evaluators were blinded to the group allocation of intervention assignments throughout the study. Detailed information on the randomization, assignment concealment, and blinding methods is described in the previous article^39^.

**Figure 3:**
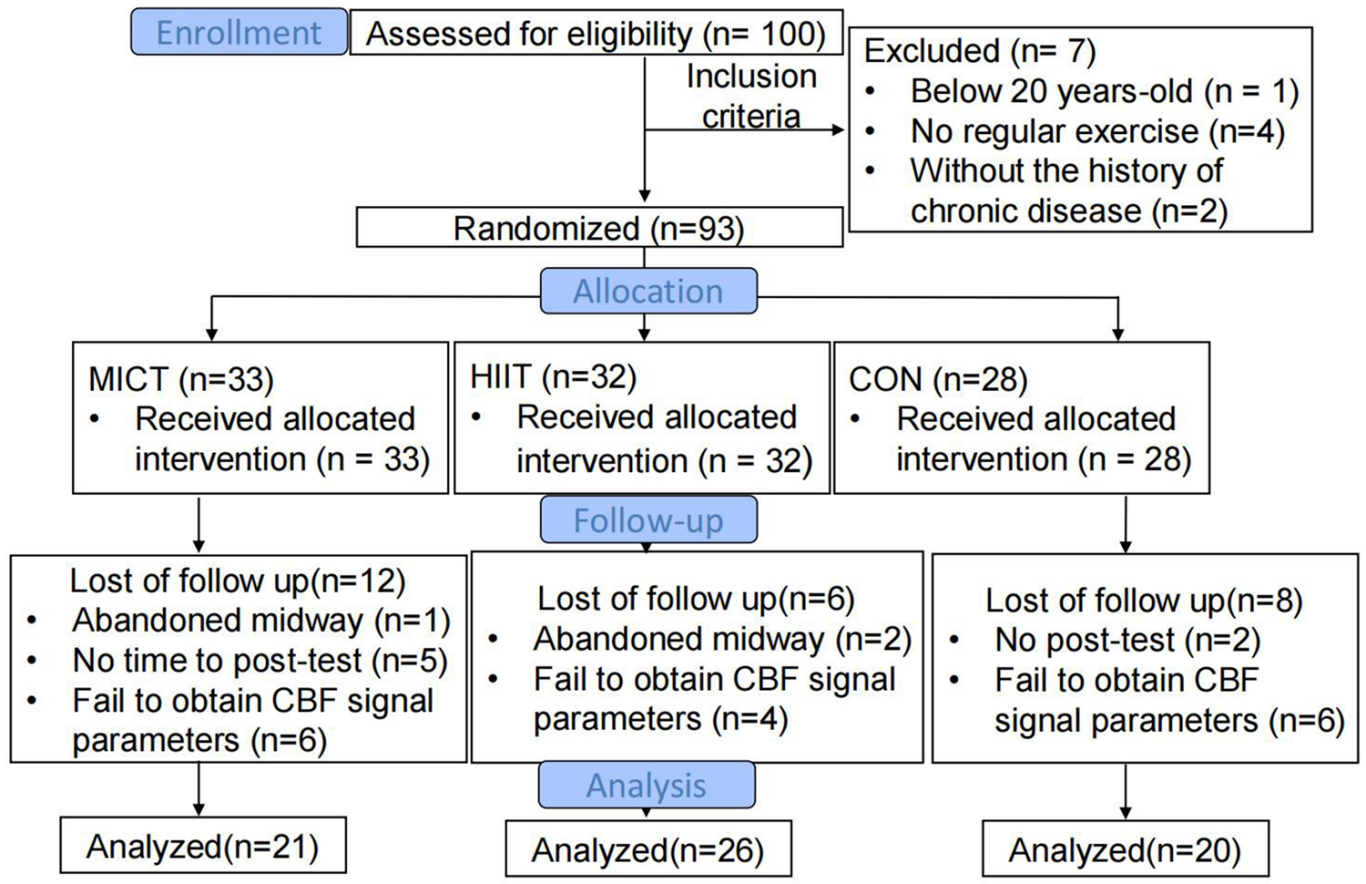
The CONSORT flow diagram. *MICT* moderate-intensity continuous training, *HIIT* high-intensity interval training, *CON* control, *CBF* cerebral blood flow.

The sample size was calculated by the software of G-Power (Version 3.1.9.7). The effect sizes of exercise interventions on cognition ranges from 0.19 to 0.59 according to the previous studies^12^. For the repeated measures one-way analysis of variance (ANOVA) test, we assumed that the effect size of the exercise intervention was 0.4 (Cohen’s f = 0.40), 0.05 for “α error probability,” and 0.8 for “power (1 − β error probability)”^40^. Moreover, the study included three groups (MICT, HIIT, and control group). According to the G-Power software, a total sample size of 64 is needed in this study. Therefore, 100 individuals were initially recruited for this study, of whom 93 were found to meet the inclusion criteria.The trial profile is provided in Fig. 3. The inclusion criteria for this study were as follows: (1) healthy young college students aged 20–30 years with normal BMI, who reported no history of cardiovascular, cerebrovascular, or respiratory disease and were not taking any cardiovascular medications; (2) individuals with normal or corrected vision; (3) participants who reported being physically inactive in the past six months, as determined by the International Physical Activity Questionnaire screening; and (4) individuals without musculoskeletal conditions that could impede participation in physical activity^39^.

This study was conducted in accordance with the International Conference on Harmonization guidelines for Good Clinical Practice and the Declaration of Helsinki. The academic ethics committee of Tsinghua university approved this study (IRB 20190091). All participants provided written informed consent prior to participation.

## Procedures and interventions

### Procedures

One week before the formal experiment after the group assignment, the participants were informed to wear comfortable sportswear and shoes to the laboratory for a VO_2max_ test to determine the individual intensity of exercise training. The outcomes were assessed at the beginning of the first visit and on the second day of the final visit of the interventions. Specifically, on the first day of the intervention, all the participants completed the baseline assessment of the outcomes (pre-test). After that, they performed different types of exercise interventions under the guidance and supervision of two professional coaches. The HIIT and MICT groups were intervened three times a week for 12 weeks, and the control group visited two times a week for the same intervention period. The outcomes were repeatedly measured on the second day morning of the final visit (post-test). To minimize the impact of biological rhythms and dietary factors, participants were instructed to fast for 12 h before the two measurements(pre-test and post-test) and to abstain from eating after 8:00 PM on the preceding night. They were advised to sleep before 11:00 PM the night before to ensure adequate rest. All the measurements were scheduled in the morning between 8:00 AM and 12:00 PM. In the two repeated evaluations before and after the intervention, all the outcomes were measured by the same evaluator, respectively, to prevent measurement errors. Additionally, we conducted an assessment to identify any significant life events that may have influenced the participants’ condition, such as the loss of significant individuals, academic or work-related failures, and other significant setbacks. All the outcomes were measured in the laboratory, and the interventions were completed in the university’s fitness centre. To ensure completion of the experiment, participants were provided with appropriate compensation, including 200 RMB and other gift rewards. The schematic diagram of experimental procedures is described in Supplementary Fig. S1.

#### Interventions

The individual intensity of exercise interventions was determined by the maximum oxygen uptake for each participant. VO_2 max_ was proven to be more accurate in determining the individual intensity of exercise intervention^41^. Cosmed Fitmate metabolic system (Cosmed, Rome, Italy) was used to obtain the parameters of oxygen consumption and VO_2 max_ when the participants performed Bruce’s treadmill protocol^42^. The participants ran on the treadmill at a fixed speed and then gradually increased until they met the following criteria: (1) felt exhausted and unable to continue running anymore, with a score of RPE ≥ 19; (2) had symptoms of dyspnea, dizziness, extreme fatigue, or pale face. (3) arrhythmia; (4) The respiratory quotient was greater than or equal to 1.1^39,43^.

We developed the training protocol referring to the previous studies^44–46^. The MICT group participants were instructed to run for 20 min at a running speed equivalent to 70–75% of their individual VO_2 max_. The HIIT group participants were directed to perform a running exercise protocol consisting of one minute at a speed equivalent to 100% of their individual VO_2 max_, followed by one minute of running at a speed equivalent to 50% of their individual VO_2 max_. This protocol was repeated ten times, with a total exercise duration of 20 min^39^. To monitor the real-time running speed, participants wore a heart rate band (Polar H10, Finland) on their chest. A 10-min warm-up and cool-down were performed before and after the 20-min intervention of HIIT and MICT, respectively, resulting in a total intervention time of 40 min. The control group participants were provided with health education lessons that did not involve any exercise. The health education primarily focused on four aspects: (1) assessment of body posture; (2) techniques for adjusting physical posture; (3) methods for stretching and relaxation; and (4) evaluation of body composition^39^.

### Primary outcomes

#### Executive function

The executive function was measured by the accuracy and reaction time of the Trail Making Test (TMT) based on the computer task, which is widely used among young adults because of its high reliability and validity^47,48^. The screen presented 13 disordered numbers from 1 to 13 and letters from A to L at random. The participants needed to alternately connect the numbers and letters in order quickly and accurately (e.g., 1 with A, A with 2, and 2 with B). The reaction time and accuracy of the TMT were collected from all participants before and after the intervention.

#### Blood flow velocity

The diastolic and systolic CBF velocities of the participants’ middle cerebral artery of the brain were measured by a transcranial Doppler (TCD) flow analyzer (EMS-9WA) before and after interventions^49^. TCD ultrasound is a non-invasive and cost-effective sensing method. It measures cerebral blood velocities with high temporal resolution^50^. Vs, the velocity of end-diastolic (Vd), and the velocity of mean cerebral blood flow (Vm) of the participants were detected using a 2 MHz TCD probe over their temporal window. Insonation of the middle cerebral artery(MCA) was confirmed using established criteria, including the position and orientation of the probe, insonation depth, and direction of blood flow about the probe^51^. All repeated measurements within each participant were taken by a single, trained investigator at the same probe depth and position to ensure the recapture of the same cerebral artery. In addition, PI was calculated using the following formula: PI = (Vs − Vd)/Vm, as a measure of the downstream resistance in the cerebral arteries^52^. RI and S/D were calculated as RI = (Vs − Vd)/Vs, S/D = Vs/Vd.

### Secondary outcomes

#### The feeling of subjective fatigue and enjoyment of exercise

We also measured the subjective feeling and enjoyment of HIIT and MICT before and after the acute exercise on the first day of the intervention. Participants’ fatigue feeling and exertion degree were recorded by the rating of RPE^53^. Studies have shown that RPE is highly believable and widely used to reflect the subjective feeling in the exercise process regarding strength, tension, fatigue, or other discomforts. Meanwhile, the PACES was employed to evaluate the subjective degree of enjoyment in carrying out physical activity. Higher scores reflect greater levels of enjoyment^54^. As the secondary outcome, we assessed the PACES of the participant during the first visit of the exercise intervention.

#### Blood pressure

Blood pressure (BP) and resting heart rate were measured using an automatic sphygmomanometer (YE670A, China). Blood pressure and resting heart rate of all participants were measured on the first day of the intervention in the morning and then repeatedly after the 12-week intervention. They were asked to sit quietly for 10 min before the test when they came to the lab. The measurement was performed twice, and the mean value was used to represent their blood pressure.

### Statistical analyses

We conducted a descriptive analysis to obtain the frequency and percentage for categorical variables and mean and standard deviation (SD) for continuous variables. We used a one-way analysis of variance (ANOVA) for continuous variables and the chi-square test for categorical variables to determine differences between groups at baseline. Given the study's repeated measurement design, we utilized mixed-effect models with a random effect on individuals nested within the group to account for intrapersonal variation (R-packages: lme4). In this study, we employed a mixed-effect model that comprised the fixed effect of groups (control, MICT, and HIIT) and measurement time (pre and post-test), while controlling for gender, age, education level, residence, self-perceived income level, and body mass index (BMI) as covariates. The interaction effect of measurement time and groups indicated the changes in group differences from pre to post-test, which indicated the causal effect of the intervention. Pairwise comparisons of changing outcomes between groups during the intervention period were further performed (R-packages: emmeans). The generalized additive model with penalized spline was used to estimate the possible nonlinearity dose–response correlations between the performance of TMT response and CBF controlling the measurement time, education, birthplace, age, and BMI. Moreover, the independent sample t-test was used to compare PACES and RPE results among HIIT and MICT groups.

We performed a series of sensitivity analyses to assess the robustness of our findings: (1) We reran the main analysis using repeated measures analysis of variance; (2) we also re-analyzed the association of CBF and executive function using a generalized linear model (Supplementary Table S6). All data analysis and visualization were conducted using R software (Version 4.1.2), and a *p-*value less than 0.05 were considered statistically significant.

## Discussion

In the 12-week double-blinded RCT study, we investigated the effect of HIIT and MICT on executive function in young adults and explored the potential underlying mechanism of exercise on cognitive performance through CB. Our findings revealed that cognitive performance improved significantly in both the HIIT and MICT groups, with MICT showing greater benefits. These results suggest that even in young adults with peak cognitive ability, exercise, especially MICT, can enhance executive function. We also observed a significant improvement in CBF, as evidenced by increased PI, RI, and S/D, in the MICT group after the 12-week intervention. Furthermore, we identified a significant association between CBF and executive function, suggesting that CBF may represent a potential mechanism through which exercise enhances executive function.

After a 12-week intervention, we observed a significant improvement in executive function among both the HIIT and MICT groups in young adults, with MICT demonstrating greater benefits. These findings are consistent with a previous study that investigated the effects of 12 weeks of combined aerobic and resistance exercise on neurocognitive performance in obese women. The study reported improvements in physical fitness, weight status, and cognitive interference inhibition following the intervention^55^. Furthermore, previous studies have demonstrated that long-term MICT and HIIT can enhance executive function in healthy elderly individuals and those with Alzheimer’s disease, which is in line with our finding^10,56^. Previous research has reported that six months of MICT (three days per week of 20–40 min at 30–70% heart rate reserve) augmented executive function in older adults, and this improvement has been associated with an enhancement in cerebrovascular regulation^57^. Additionally, chronic exercise has been found to have positive effects on the executive function of overweight children^58^. However, a previous study suggested that improving executive function may be more challenging in younger populations, as there may be less room for improvement^22^. Thus, our findings have expanded the population that may benefit from exercise on cognitive performance.

We sought to explore whether different exercise modalities (MICT and HIIT) had comparable effects on enhancing executive function in young adults. Our results demonstrated that the MICT intervention was more effective than HIIT after 12 weeks of interventions. This finding is consistent with a previous article, which reported that aerobic exercise, such as MICT, can create a nutritive environment in the brain by facilitating cortical activity, hemodynamics, and metabolism^59^. However, in older adults, evidence suggests that HIIT elicits more significant blood flow responses and fluctuations in metabolic intensity compared to MICT, leading to more remarkable changes in cerebrovascular function after six weeks of intervention^60^. These beneficial effects could translate to favourable improvements in higher-order cognitive functions^60^. HIIT seems to provide greater beneficial effects by increasing the amount of neurotrophic factor^61,62^. However, the inconsistent findings of previous studies on the effects of HIIT and MICT interventions may be attributed to variations in study populations and intervention duration. Additionally, prolonged HIIT may lead to fatigue, decreased cerebral oxygenation, and elevated blood lactate levels, which may reduce its facilitative effects^63^. Our study provides guidance for young adults to choose appropriate exercise modalities to enhance executive function and maintain brain health in real-life settings.

Following the 12-week intervention, statistically significant differences were observed in PI, RI, and S/D of the CBF, particularly in MICT group. Research has demonstrated that changes in CBF are intensity-dependent, with CBF velocity in the MCA increasing linearly with exercise intensity until approximately 60–70% of maximal VO_2 max_, as reported in previous studies^64,65^. Regarding the long-term effect of exercise on CBF, a previous longitudinal study observed an age-related decline in middle cerebral artery blood velocity (MCAv), which was consistently elevated in endurance-trained men across all age ranges^66^. Additionally, previous research has reported that a 12-week aerobic exercise training program significantly increased MCAv in both young and older participants^67^. The PI of the cerebral artery is widely regarded as an indicator of downstream arterial resistance^68^. Sympathetic nervous system activation during exercise has been reported to increase cerebral PI^69^. Consistent with prior findings, our study revealed an elevated PI value at rest, which may contribute to the increased Vs observed in the exercise groups^70^. A previous study also reported an elevation in MCAv and a reduction in resting heart rate following 12-week training, suggesting similar physiological adaptations in response to exercise^70^. However, the underlying mechanisms responsible for the observed changes in Vs in healthy young adults require further investigation.

Several studies have reported the effect of exercise on cognitive function, often focusing on changes in brain activation and synaptic plasticity assessed using fMRI and EEG in humans^71^. However, there is a lack of evidence regarding the underlying CBF mechanisms that contribute to the exercise-induced improvement of executive function. In this study, we examined the effect of exercise on CBF and found a significant association between CBF and executive function in young adults, providing insights into the CBF mechanisms underlying the exercise-cognition relationship. Consistent with our findings, animal studies have shown that long-term exercise induces angiogenesis and neurogenesis in the brain, leading to increased cerebral perfusion and metabolism^72,73^, which manifests as increased cerebral perfusion and metabolism^74^. In middle-aged animals, aerobic exercise also enhances vascular growth factor production and promotes angiogenesis, supporting optimal cerebrovascular functioning^75,76^, These findings may explain the link between exercise training, CBF, and higher executive function^59^.

Some limitations should be acknowledged in this study. Firstly, 12 weeks of intervention is relatively short for improving young adults’ CBF and executive function. Future studies with extended intervention periods are preferred to further explore the benefits. Secondly, our sample was small considering the methodological limitations, such as the challenges of CBF assessment with transcranial Doppler ultrasonography due to the thickness of the temporal bone, which even resulted in some participants from whom no signal could be acquired at all. However, all the CBF indexes were assessed by the same researcher and measured in a similar way during the whole process of the research. Additionally, we checked and confirmed data quality during preprocessing to ensure the measurement validity of CBF. Thirdly, the intermediary effect of CBF was not found, which may be due to the small sample size. We were able to identify a significant association between CBF and executive function. Thus, well-designed randomized controlled trials with larger sample sizes are needed to assess the effects of exercises on executive function and the mediation mechanism of CBF. Finally, the RCT study was conducted in a population of healthy young adults, so the study findings may not be generalizable to older people and clinical patients. However, this study also has notable strengths. First, to the best of our knowledge, this is the first double-blind RCT study that provides direct  evidence comparing the effects of MICT and HIIT interventions on executive function in young adults. Secondly, to determine the appropriate MICT and HIIT protocol, an individual’s maximal oxygen uptake was measured through a laboratory cardiopulmonary exercise test. This approach is considered more precise than other methods employed in previous studies. Most importantly, this randomized controlled trial study provides valuable insights into the causal effects of MICT/HIIT intervention on executive function. Moreover, the evidence will be necessary for understanding why exercise can improve executive function and further confirm the CBF mechanism of exercise on executive function. The results provided suggestions to young adults when choosing different modalities of exercise in real life to improve executive function and maintain brain health.

## Conclusions

This study is the first to demonstrate that a 12-week MICT intervention improves CBF and executive function to a greater extent than HIIT in young adults. Moreover, our findings suggest that CBF is a potential mechanism underlying the exercise-induced enhancement of executive function in young individuals, providing practical evidence to encourage regular exercise for maintaining cognitive function and promoting brain health.

## Supplementary Information

Supplementary Information.

## Data Availability

The dataset used and/or analysed during this current study are not publicly available as the raw dataset has incorporated with participants identifiable information, but are available from the corresponding author on reasonable request.
