# Supplementary material for: The effect of exercise on cerebral blood flow and executive function among young adults: a double-blinded randomized controlled trial
Source: Sci Rep. 2023 May 22;13:8269. doi: 10.1038/s41598-023-33063-9 (PMC10203129; doi:10.1038/s41598-023-33063-9)
Supplement: Supplementary file 1 — Supplementary Information. [file 41598_2023_33063_MOESM1_ESM.docx]

**The Effect of Exercise on Cerebral Blood Flow and Executive Function among Young Adults: A Double-blinded Randomized Controlled Trial**

Jianxiu Liu, Ph.D.^1, 2, †^, Leizi Min, M.S.^2 †^, Ruidong Liu, Ph.D.^3†^, Xiaoyu Zhang, M.S.^4^, Meiting Wu, M.S.^5^, Qian Di, S.c.D^1,6 *^, Xindong Ma, Ph.D.^2, 7*^

1. Vanke School of Public Health, Tsinghua University, Beijing, China, 100084, liujianxiu@mail.tsinghua.edu.cn

2. Division of Sports Science and Physical Education, Tsinghua University, Beijing, China, 100084, mlz20@mails.tsinghua.edu.cn

3. Sports Coaching College, Beijing Sport University, Beijing, China, 100084, lrd5156@bsu.edu.cn

4. AME2P Laboratory, Clermont Auvergne University, Clermont-Ferrand, France, 63178, xiaoyuzhang_0714@163.com

5. Department of Physical Education, Zhejiang College of Sports, Hangzhou City, Zhejiang, China, 310013, wumeitinghhxx@163.com

6. Institute for Healthy China, Tsinghua University, Beijing, China, 100084, qiandi@tsinghua.edu.cn

7. IDG/McGovern Institute for Brain Research, Tsinghua University, Beijing, China, 100084, maxd@mail.tsinghua.edu.cn

†These authors contributed equally to this work

* Equal corresponding authors

Corresponding author: Vanke School of Public Health, Tsinghua University, Beijing 100084, China, qiandi@tsinghua.edu.cn; Division of Sports Science & Physical Education, Tsinghua University, Beijing 100084, China, maxd@mail.tsinghua.edu.cn.

**Table S1 The effect of acute exercise on RPE and PACES**

|  | **MICT（N=21）** | **HIIT（N=26）** | **t** | ***P*** |
| --- | --- | --- | --- | --- |
| **RPE** | 13.485 | 12.724 | -1.004 | 0.320 |
| **PACES** | 59.939 | 66.828 | 1.093 | 0.279 |

***Notes:*** MICT: moderate-intensity continuous training; HIIT: high-intensity interval training; RPE: the rating of perceived exertion scale; PACES: the physical activity enjoyment scale. The effects were estimated by the independent-sample t-test.

**Table S2 Results of mixed effect models: Changes in blood pressure and resting heart rate from pre- to post-measurement.**

| Variable | Group ^a^  β (95% CI) | | Time ^b^  β (95% CI) | Group*Time ^c^  β (95% CI) | |
| --- | --- | --- | --- | --- | --- |
|  | MICT | HIIT |  | MICT*post | HIIT*post |
| Systolic pressure (mmHg) | 4.41(-2.558, 11.379) | 4.879(-1.902, 11.659) | **7.84 (3.478, 12.201) **** | **-8.931(-15.633, -2.229) *** | **-11.057(-17.419, -4.695) **** |
| Diastolic pressure (mmHg) | 3.209(-1.14, 7.557) | 2.275(-1.967, 6.517) | 1.682 (-0.926, 4.289) | **-4.952(-8.972, -0.931) *** | **-4.474(-8.282, -0.666) *** |
| Rest heart rate (times/minute) | -0.135(-6.277, 6.007) | -1.652(-7.586, 4.282) | 4.222 (-0.190, 8.634) | **-7.353(-14.037, -0.669) *** | **-7.687(-14.132, -1.242) *** |

Note βeffect estimate; CI, confidence interval; MICT, moderate-intensity continuous training; HIIT, high-intensity interval training. Results were calculated using mixed-effect models with a random effect on individuals to account for intrapersonal variation.

a Reference category: Control group

b Reference category: Pre measurement

c Differences between the MICT and control group or the HIIT and control group from pre- to post-measurement, indicating the intervention effect.

* *p* < 0.05, ** *p* < 0.01

**Table S3 The coefficient of variation of the CBF**

|  | **The coefficient of variation** | |
| --- | --- | --- |
|  | **Pre-test** | **Post-test** |
| **Vs** | **14.92%** | **15.11%** |
| **Vm** | **15.60%** | **15.53%** |
| **Vd** | **17.97%** | **18.49%** |
| **PI** | **13.87%** | **17.21%** |
| **RI** | **9.00%** | **10.24%** |
| **S/D** | **10.75%** | **15.55%** |

***Notes*:** Vs: velocity of peak-systolic; Vd: velocity of end-diastolic; Vm: velocity of mean cerebral blood flow; PI: pulsatility index; RI: resistance index; S/D: peak-systolic of cerebral blood flow/end-diastolic of cerebral blood flow.

**Table S4 The estimated effect of interventions on executive function and CBF among three groups at pre and post measurements**

| **Variables** | **Group** | **Estimated values ^a^** | ***p*** |
| --- | --- | --- | --- |
| **Time** | **MICT** | -17.30(-24.33, -10.33) | **<.0001** |
|  | **HIIT** | -10.70(-17.06, -4.48) | **0.001** |
|  | **Control** | -7.20(-0.19, 14.51) | 0.061 |
| **Accuracy** | **MICT** | 0.04(0.01, 0.08) | **0.016** |
|  | **HIIT** | 0.03(-0.06, 0.01) | 0.105 |
|  | **Control** | 0.002(-0.03, 0.04,) | 0.910 |
| **Vs (cm/s)** | **MICT** | 3.10(-2.90, 9.10) | 0.315 |
|  | **HIIT** | 1.70(-3.66, 7.12) | 0.531 |
|  | **Control** | -1.10 (-7.33, 4.93) | 0.703 |
| **Vm (cm/s)** | **MICT** | 0.10(-4.12, 4.31) | 0.965 |
|  | **HIIT** | 0.001(-3.80, 3.80) | 1.000 |
|  | **Control** | -0.10(-4.48, 4.18) | 0.946 |
| **Vd (cm/s)** | **MICT** | -1.60(-5.40, 2.16) | 0.404 |
|  | **HIIT** | -1.00(-4.35, 2.43) | 0.581 |
|  | **Control** | 0.55(-3.31, 4.41) | 0.781 |
| **PI** | **MICT** | 0.10(0.03, 0.17) | **0.010** |
|  | **HIIT** | 0.04(-0.03, 0.10) | 0.238 |
|  | **Control** | -0.02(-0.10, 0.05) | 0.521 |
| **RI** | **MICT** | 0.03(0.01, 0.06) | **0.018** |
|  | **HIIT** | 0.02(-0.01, 0.04) | 0.229 |
|  | **Control** | -0.01(-0.04, 0.02) | 0.481 |
| **S/D** | **MICT** | 0.23(0.06, 0.39) | 0.008 |
|  | **HIIT** | 0.08(-0.06, 0.23) | 0.274 |
|  | **Control** | -0.06(-0.22, 0.11) | 0.537 |

***Notes*:** a indicated the adjusted mean difference of pre and post. Vs: velocity of peak-systolic; Vd: velocity of end-diastolic; Vm: velocity of mean cerebral blood flow; PI: pulsatility index; RI: resistance index; S/D: peak-systolic of cerebral blood flow/end-diastolic of cerebral blood flow.

**Table S5 The associations between executive function and CBF estimated by generalization additive model**

| **Variables** | **Group** | **F** | ***p*-value** |
| --- | --- | --- | --- |
| **Time^a^** | **Vs** | 5.414 | 0.022 |
|  | **Vm** | 2.224 | 0.075 |
|  | **Vd** | 2.434 | 0.136 |
|  | **PI** | 4.973 | 0.012 |
|  | **RI** | 5.845 | 0.006 |
|  | **SD** | 3.168 | 0.063 |
| **Accuracy** | **Vs** | 2.300 | 0.143 |
|  | **Vm** | 0.117 | 0.832 |
|  | **Vd** | 0.535 | 0.466 |
|  | **PI** | 4.797 | 0.036 |
|  | **RI** | 5.394 | 0.024 |
|  | **SD** | 4.312 | 0.050 |

***Notes*:** CBF: cerebral blood flow; a indicated the total time of the trail-making test; b indicated the accuracy of the trail-making test. Vs: peak-systolic; Vd: end-diastolic; Vm: mean cerebral blood flow velocities; PI: pulsatility index; RI: resistance index; S/D: peak-systolic of cerebral blood flow/end-diastolic of cerebral blood flow.

**Table S6 Sensitive analysis of the associations between executive function and CBF using generalization linear model**

| **Variables** | **Group** | **β (95% CI)** | ***p*-value** |
| --- | --- | --- | --- |
| **Time** | **Vs** | -0.288(-0.5, -0.076) | 0.009 |
|  | **Vm** | -0.334(-0.667, -0.001) | 0.051 |
|  | **Vd** | -0.227(-0.637, 0.184) | 0.282 |
|  | **PI** | -16.243(-38.274, 5.788) | 0.151 |
|  | **RI** | -50.813(-108.165, 6.54) | 0.085 |
|  | **SD** | -5.711(-15.757, 4.336) | 0.267 |
| **Accuracy** | **Vs** | 0.0003(-0.0004, 0.001) | 0.379 |
|  | **Vm** | 0.0001(-0.001, 0.001) | 0.908 |
|  | **Vd** | -0.001(-0.002, 0.001) | 0.466 |
|  | **PI** | 0.091(0.019, 0.164) | 0.015 |
|  | **RI** | 0.255(0.065, 0.444) | 0.01 |
|  | **SD** | 0.039(0.006, 0.072) | 0.023 |

***Notes*:** CBF: cerebral blood flow; a indicated the total time of the trail-making test; b indicated the accuracy of the trail-making test. Vs: velocity of peak-systolic; Vd: velocity of end-diastolic; Vm: velocity of mean cerebral blood flow; PI: pulsatility index; RI: resistance index; S/D: peak-systolic of cerebral blood flow/end-diastolic of cerebral blood flow.


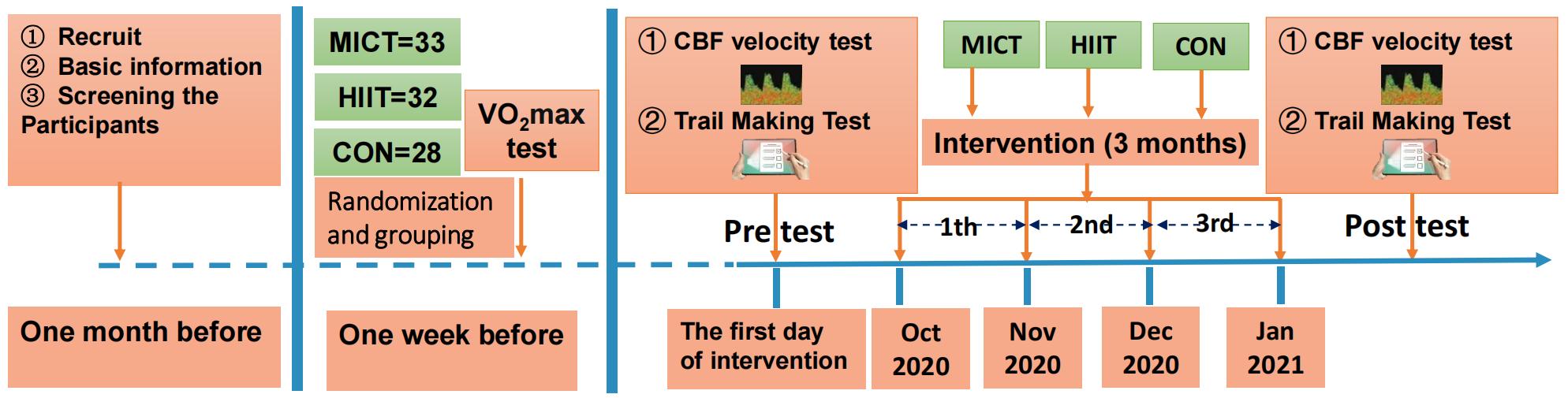


**Figure S1. The Schematic Diagram of Experimental Procedures**

***Notes:* MICT: moderate-intensity continuous training; HIIT: high-intensity interval training; CON: control; CBF: cerebral blood flow**
